# Why We Need More Nature at Work: Effects of Natural Elements and Sunlight on Employee Mental Health and Work Attitudes

**DOI:** 10.1371/journal.pone.0155614

**Published:** 2016-05-23

**Authors:** Mihyang An, Stephen M. Colarelli, Kimberly O'Brien, Melanie E. Boyajian

**Affiliations:** 1 The School of Public Service and Global Citizenship, Central Michigan University, Mount Pleasant, MI, United States of America; 2 Department of Management, School of Business, Hong Kong Baptist University, Hong Kong, China; 3 Department of Psychology, Central Michigan University, Mount Pleasant, MI, United States of America; Istituto Superiore di Sanità, ITALY

## Abstract

This study investigated the effects of natural elements and direct and indirect sunlight exposure on employee mental health and work attitudes. We recruited participants via an online panel from the United States and India, and analyzed data from 444 employees. Natural elements and sunlight exposure related positively to job satisfaction and organizational commitment, and negatively to depressed mood and anxiety. Direct sunlight was a dominant predictor of anxiety; indirect sunlight was a dominant predictor of depressed mood, job satisfaction, and organizational commitment. Natural elements buffered the relationship between role stressors and job satisfaction, depressed mood, and anxiety. We also found that depressed mood partially mediated the relationship between natural elements and job satisfaction. We discuss scientific and policy implications of these findings.

## Introduction

Work in modern settings has been epidemiologically linked to a variety of maladies. Many of these are stress-related illnesses—such as depression, anxiety, hypertension, and gut ailments; others—lower back pain, some sleep disorders, and respiratory problems—are more related to physical conditions [1]. Much of the research on worker health, particularly mental health and other stress-related diseases, has focused on improved management practices (e.g., giving employees more control over their work and schedules; improving interpersonal skills) and palliative stress-reduction treatments (e.g., meditation, mindfulness training, and employee fitness centers) [2, 3]. Interestingly, little organizational and management research has examined the effects of the physical work environment itself on employees [4, 5]. However, it is important to consider the work environment as a causal and remedial factor in employee health. People spend a great deal of time at work. In fact, adults spend about 40 hours per week in offices, most of the time at desks and workstations [6]. Research (mainly from non-management disciplines) on physical characteristics of workplaces (e.g., lighting, noise, air quality) shows that the physical characteristics can influence employee health [7]. Considerable research also exists on the effects of *natural environments*–exposure to natural elements (such as greenery) and sunlight—on physical and mental health [8–16]. However, few studies have examined the effects of natural environments in work settings [12–13, 17]. Unlike improved management practices and palliative stress reduction programs, the physical work environment can be a continuous health promoting intervention—one that requires neither extra effort from employees nor dedicated time. This study focuses on the effects of exposure to natural elements and sunlight on employee mental health and job attitudes. We also look at the unique and combined effects of natural elements and sunlight.

### The Work Environment and Exposure to Natural elements and Sunlight

Organizations consist of individuals and groups engaged in collective action to pursue common goals. As such, organizations require resources to survive and prosper. Organizational resources include people, energy, material, technology, knowledge, and capital [18]. Ironically, organizations have paid relatively little attention to natural elements and sunlight—two resources that are available to almost all organizations, that are free or relatively inexpensive, and that can provide physiological and psychological benefits to employees [5].

The influence of Taylorism may be partially to blame for this lack of attention. The design of most modern workspaces still focuses on space efficiency—to maximize work flow and minimize space costs per employee [11]. This approach typically excludes materials not directly related to the tasks at hand [19], resulting in barren and angular work spaces. Another culprit may include beliefs about human malleability. A common belief among managers and management scholars is that people are malleable and therefore can be socialized to work in almost any condition [20]. This belief would encourage designs maximizing efficiency, with little attention paid to human needs.

#### Natural elements exposure

Exposure to natural elements is associated with decreased levels of diastolic blood pressure, depression, and anxiety [9, 11, 21], and increased attentional capacity [22]. Exposure to natural elements (e.g., green spaces) can reduce the impact of stress [23], increase psychological well-being [14, 15, 24], and support recovery from illness [14, 25]. Compared to people exposed to urban views, those exposed to views of nature discount the future less. That is, they place a greater value on the future, which has consequently been associated with a healthier lifestyle [26].

One explanation for the link between natural elements exposure and improved mental health involves mechanisms that reduce mental fatigue. Natural elements have a restorative effect on mental fatigue [9, 27]. Attention-restoration theory suggests that the mind is like a muscle [28]. When the mind is engaged in *directed* attention—as would be the case when it is exposed to built environments and after extended concentration on work tasks—it becomes fatigued and requires rest and recuperation to function effectively. Exposure to nature involves indirect attention, characterized by fascination [29]. This has a restorative effect on the mind, countering fatigue—much like rest has on a fatigued muscle.

Another explanation is that exposure to natural elements has a calming effect on our physiology. Ulrich and his colleagues argue that natural elements are evolved, unconditioned stimuli, associated with environments that were typically safe and resource-rich in our evolutionary past [30]. Just as snakes, spiders, and heights are evolved, unconditioned stimuli producing fear, natural elements (such as greenery, savannah-like landscapes, and clear running water) have an automatic calming effect on physiological arousal. Thus, in work settings with stress-producing stimuli (e.g., role conflict, role ambiguity, time demands, and heavy workload), exposure to natural elements has calming effects. In comparing regions of the brain activated when viewing pictures of rural and urban environments, the hippocampus (a locus for memory) and amygdala (a locus for anger and fear) are activated when people view urban scenes—suggesting increases in working memory and arousal of stressful emotions [31]. Rural scenery activates the basal ganglia, a region of the brain that is associated with pleasure [31].

Many of the benefits of natural elements can result from direct (e.g., plants in the office), indirect (e.g., window views), or representational (e.g., photographs) exposure to natural elements [12, 32, 33]. Exposure to plants can improve mood, reduce stress, and detoxify office air [34, 35]. Representations and window views can also be beneficial; interestingly, photographs or paintings of nature in an office setting seem to have similar effects as views of nature through a window [36].

Research on the benefits of natural elements exposure is compelling, and therefore we expect that natural elements exposure in the workplace will be positively related to employee mental health. Because exposure to natural elements is a valued resource, it should be positively associated with job attitudes as well. When employees obtain valued resources, they experience less discomfort [37] and are more likely to have positive attitudes toward their jobs and organizations [38, 39].

Hypothesis 1: Exposure to natural elements in the workplace will be positively related to employees’ mental health (lower depressed mood and anxiety) and work attitudes (higher job satisfaction and organizational commitment).

Although we expect a positive relationship between natural elements and job satisfaction, as Dravigne and colleagues [40] have found, we believe that the relationship may be somewhat more complex and mediated by mood. Job satisfaction is an *emotional* reaction to the job and therefore it is likely to be influenced by factors affecting employee mood. Because natural elements influence mood [41], it is plausible that the relationship between natural elements and job satisfaction is mediated by depressed mood. In other words, we expect that a lack of natural elements exposure will lead to depressed mood, which in turn will reduce job satisfaction.

Hypothesis 2: The relationship between natural elements and job satisfaction is mediated by depressed mood.

Employees inevitably encounter stressors at work. Role stressors are commonly related to lower mental health and poor job attitudes [37, 42]. Because environments with exposure to natural elements exert restorative effects on mental fatigue and physiological arousal, these environments should mitigate the relationship between role stressors and employee mental health and work attitudes [43]. That is, the effects of stressors on employees should be less severe (buffered) when employees are in environments with greater levels of exposure to natural elements.

Hypothesis 3a, b: Exposure to natural elements will moderate the relationship between role stressors and employee mental health and work attitudes. Specifically, for individuals who report more natural elements exposure at work, the relationships between (a) role stressors and mental health (i.e., depressed mood and anxiety) and (b) between role stressors and job attitudes (i.e., job satisfaction and organizational commitment) will be weaker than for the employees who report lower levels of natural elements exposure.

#### Sunlight exposure

Direct and indirect sunlight are important resources for physical and mental health. *Direct* sunlight refers to sunlight exposure while outside without any interference. *Indirect* sunlight, on the other hand, refers to refracted sunlight, which could be, for example, sunlight exposure through windows. Direct sunlight exposure to the skin stimulates vitamin D synthesis. Vitamin D improves the immune function, regulates the inflammatory response, and influences calcium homeostasis [10, 44]. Direct and indirect sunlight exposure also influence the sleep cycle [45]. Direct and indirect sunlight exposure on the retina stimulate intrinsically sensitive retinal ganglion cells (isRGC), thereby influencing the secretion of melatonin, which is critical for regulating the sleep-wake cycle (circadian rhythm) [45]. Direct and indirect sunlight also influence the production of serotonin [46], a neurotransmitter that elevates mood [47]. Sunlight (bright light in general) also influences alertness and vitality [48, 49]. Bright light (with its shorter wave lengths) affects endocrine and neurophysiological responses in the brain that trigger alertness [50].

All of the above suggest that exposure to sunlight at work should be related to employee mood. As with exposure to natural elements, we expect that exposure to sunlight in the workplace will be positively related to employee mental health. Another similarity with natural elements exposure is that sunlight exposure is a valued resource. When employees obtain valued resources, they experience less stress and are more likely to have positive attitudes toward their jobs and organizations [37], suggesting that exposure to sunlight should also be positively related with job attitudes.

Hypothesis 4: Sunlight exposure will be associated with less depressed mood and anxiety and greater job satisfaction and organizational commitment.

### Disentangling the Effects of Natural elements and Sunlight

Exposure to natural elements frequently involves exposure to sunlight. As a result, the effects of natural elements and sunlight can be conflated [51]. Most studies typically focus on one or the other—natural elements [30] or sunlight [52]. However, by examining natural elements and sunlight exposure simultaneously, we can parse their effects on mental health and work attitudes. Because there is little empirical overlap between our measures of natural elements and sunlight exposure, we should be able to estimate their relative independent contributions to mental health and work attitudes.

## Method

### Participants and Procedure

Participants were recruited through Amazon.com’s online marketplace, Mechanical Turk (MTurk; www.mturk.com), consistent with recommendations used in previous research [53, 54]. All participants received 75 cents as payment for their participation, as suggested by MTurk, based upon the number of items and the length of time necessary to complete the survey. Prior to data collection, this study was approved by Central Michigan University’s Institutional Review Board (approval number: 153962–2). Data were collected from a bi-national sample from the United States and India. Two distinct sources of data were used to enhance the generalizability of the results and to aid in assessing the quality of our methods. After removing unqualified participants (e.g., inattentive respondents, those working less than 20 hours per week), there were a total of 444 usable responses (70% retention rate). The sample consisted of 53.4% females, with an average age of 31 (*SD* = 9.77), 54.3% of whom were Asian, followed by 34.9% Caucasian. All participants reported being employed at the time of participation and represented various industries including business, education, training, retail, information technology, and manufacturing.

### Measures

#### Natural elements exposure

Researchers have measured exposure to natural elements in a variety of ways. Most commonly, it has been measured by “view”—the degree to which natural elements are visible, typically through windows [12, 55]. However, as noted above, the salutary effects of natural elements exposure can occur from window views of nature, depictions of nature on office walls, and immersion in natural settings. Therefore, our interest was in measuring natural elements in *general—*that is, natural elements that could be viewed directly, indirectly, or representationally, as might be typical in many workspaces. We developed a scale measuring perceived exposure to natural elements that included nine items about potted plants, photographs or paintings depicting nature, and viewing natural environments through windows or computer screen savers. Respondents were asked to indicate the extent to which they agreed to each statement, for example, “There are potted plants in my workspace” and “I am exposed to depictions of nature (painting, photograph) at my workspace (see S1 Scale).” Participants responded on a 5-point Likert scale ranging from 1, “strongly disagree” to 5, “strongly agree.” The internal consistency of the scale was .93. Confirmatory factor analyses (CFAs) showed that a single factor model was excellent fit, χ^2^ (27) = 332.63 (*p* = .00), CFI = .95, SRMR = .05 [56]. The fit indices for a multi-group CFA, with factor loadings constrained to be equal across groups, was χ^2^ (54) = 368.17 (*p* = .00) and CFI = .94, providing evidence that the relationships between items and their latent construct (natural elements) were approximately the same strength across the two different countries [57].

#### Sunlight exposure

Because direct and indirect sunlight exposure have somewhat different effects (e.g., only direct sunlight stimulates vitamin D), we distinguish between them. While sunlight exposure is commonly measured by the size of sunlight patches in a workspace [12], we believe that a more general measure of sunlight exposure is also useful because employees rarely remain in one place during the workday. They may, for example, walk outside or go to areas of their building where there may get more or less sunlight exposure. We developed scales measuring perceived amounts of direct (3 items) and indirect (5 items) sunlight exposure (see S2 Scale). The direct sunlight items represented situations where individuals were exposed to sunlight by being outside—for example, “I am exposed to direct sunlight from being outside while at work.” The indirect sunlight items asked respondents whether they were exposed to sunlight indoors—for example, “There are windows that allow for natural sunlight to come in.” All items were rated on a 5-point Likert scale ranging from 1, “strongly disagree” to 5, “strongly agree.” Results of CFA indicated that a two-factor model (i.e., direct and indirect sunlight) was adequate and a better fit (χ^2^ (19) = 258.34 (*p* = .00), CFI = .92, SRMR = .09) than a single factor model (χ^2^ (20) = 457.67 (*p* = .00), CFI = .86, SRMR = .12) [56]. The two sub-scales correlated moderately (*r* = .40, *p* < .01). The internal consistency of the full scale was .86; the direct and indirect subscales were.73 and .91, respectively.

The fit indices for a multi-group CFA, with factor loadings constrained to be equal across groups, were χ^2^ (38) = 352.31 (*p* = .00) and CFI = .89, indicating a marginal fit [58, 59] and providing evidence that participants from the two different countries showed approximately the same strength of relationships between items and their underlying construct (sunlight) [57].

#### Differentiation between natural elements and sunlight

A CFA was conducted to examine whether our measures adequately distinguished natural elements from sunlight exposure [60]. The three factor model, consisting of natural elements, direct sunlight, and indirect sunlight was an adequate and better fit to the data (χ^2^ (116) = 1090.26 (*p* = .00), CFI = .91, SRMR = .11) than two (i.e., natural elements and sunlight exposure; χ^2^ (118) = 1300.07 (*p* = .00), CFI = .89, SRMR = .12) or single factor models (χ^2^ (119) = 3801.03 (*p =* .00), CFI = .77, SRMR = .19) [56].

#### Depressed mood

The Center for Epidemiological Studies Depression (CES-D) Scale [61] was used to assess depressive symptoms in participants. The CES-D is a self-report scale, consisting of 20 questions that ask participants to rate their feelings over the past week. The internal consistency of this scale was .92.

#### Anxiety

Anxiety symptoms were measured with the Beck Anxiety Inventory [62]. This 21-question self-report scale asks participants to rate how often within the past month they have been bothered by common symptoms of anxiety such as numbness, tingling, and feelings of choking. The internal consistency of this scale was .97.

#### Job satisfaction

Job satisfaction was measured with the Cammann, Fichman, Jenkins, and Klesh scale [63], with three items that ask respondents to indicate, in general, how satisfied they are with their job along a 7-point Likert scale, ranging from 1, “strongly disagree” and 7, “strongly agree.” The internal consistency of this scale was .90.

#### Organizational commitment

Organizational commitment was measured with the Mowday, Steers, and Porter scale [64], which consists of 15 items asking respondents to indicate how they feel about their organization along a 5-point Likert scale, ranging from 1, “strongly disagree” and 5, “strongly agree.” The internal consistency of this scale was .88.

#### Role stressors

Role stressors were measured with the scales developed by Rizzo, House, and Lirtzman [65] consisting of two components: role ambiguity including 6 items (e.g., “I know exactly what is expected of me”) and role conflict including 8 items (e.g., “I have to do things that should be done differently”). They are rated along a 5-point Likert scale, ranging from 1, “strongly disagree” and 5, “strongly agree.” The internal consistency of the role stressor was .83.

#### Control variables

Due to the potential influence of demographic characteristics on the level of sunlight and natural elements exposure, as well as psychological well-being, we decided to control several demographic variables. Compared to the U.S., India is less industrialized and is classified as a middle income country according to World Bank Development criteria, with low levels of reported depression [66]. We, therefore, anticipated the existence of some regional differences between the U.S. and India. The results of multi-group CFAs for exposure to sunlight and natural elements, however, found that both the US and Indian samples had approximately the same strength between items and their latent constructs, thereby justifying merging the groups into a single sample. We controlled for sex and age based upon previous findings suggesting that depression and anxiety are related to both of these demographics [66, 67].

## Results

Descriptive statistics, reliability estimates, and correlations among variables are displayed in Table 1. Data were standardized to reduce multicollinearity and enhance interpretability.

**Table 1:** Descriptive statistics and correlations among study variables.

	Mean (SD)	1	2	3	4	5	6	7	8	9	10
**1. Sex** ^a^		-									
**2. Age**	31.10 (9.77)	.08	-								
**3. Exposure to natural elements**	3.23 (1.10)	-.06	-.11*	(.93)							
**4. Exposure to direct sunlight**	3.11 (.98)	-.15	.18**	.07	(.73)						
**5. Exposure to indirect sunlight**	3.49 (1.03)	-.09	-.02	.44**	.40**	(.91)					
**6. Role stressors**	2.44 (.53)	-.06	-.14**	-.06	-.02	-.25**	(.83)				
**7. Depression**	1.66 (.53)	-.05	-.14**	-.14**	.05	-.18**	.47**	(.92)			
**8. Anxiety**	1.58 (.75)	-.17*	-.14**	-.00	.20**	-.05	.40**	.72**	(.97)		
**9. Job satisfaction**	5.35 (1.36)	-.08	-.01	.36**	.20**	.41**	-.41**	-.40**	-.15**	(.90)	
**10. Organizational commitment**	3.42 (.64)	-.02	-.11*	.28**	.19**	.35**	-.48**	-.35**	-.17**	.71**	(.88)

Note. N = 391–444. The values in parentheses on the diagonal are internal consistency reliabilities.

^a^ coded 1 = male, 2 = female.

*p < .05,

** *p* < .01

Hypothesis 1 was tested using hierarchical multiple regression analyses, controlling for age and sex. This hypothesis stated that exposure to natural elements in the workplace would be related to employees’ mental health (lower depressed mood and anxiety) and work attitudes (higher job satisfaction and organizational commitment). The results of regression analyses indicated that natural elements exposure was negatively related to depressed mood (*β* = -.17, *p* < .01) and positively related to job satisfaction (*β* = .37, *p* < .01) and organizational commitment (*β* = .30, *p* < .01), partially supporting Hypothesis 1 (see Table 2).

**Table 2:** Effects of natural elements on outcome variables.

Criteria	Variables	Step 1 *β*	Step 2 *β*	Δ *R*^*2*^	Total *R*^*2*^
**Depression**	Sex ^a^	-.04	-.05	.02**	.05**
	Age	-.15**	-.17**		
	Natural elements		-.17**	.03**	
**Anxiety**	Sex	-.17**	-.17**	.04**	.04**
	Age	-.13**	-.14**		
	Natural elements		-.03	.00	
**Job satisfaction**	Sex	-.09	-.07	.00	.14**
	Age	.02	.06		
	Natural elements		.37**	.13**	
**Organizational commitment**	Sex	-.03	-.00	.01	.09**
	Age	.11*	.14**		
	Natural elements		.30**	.09**	

Note. N = 391–410.

^a^ coded 1 = male, 2 = female.

*p < .05,

**p < .01

**Fig 1:**
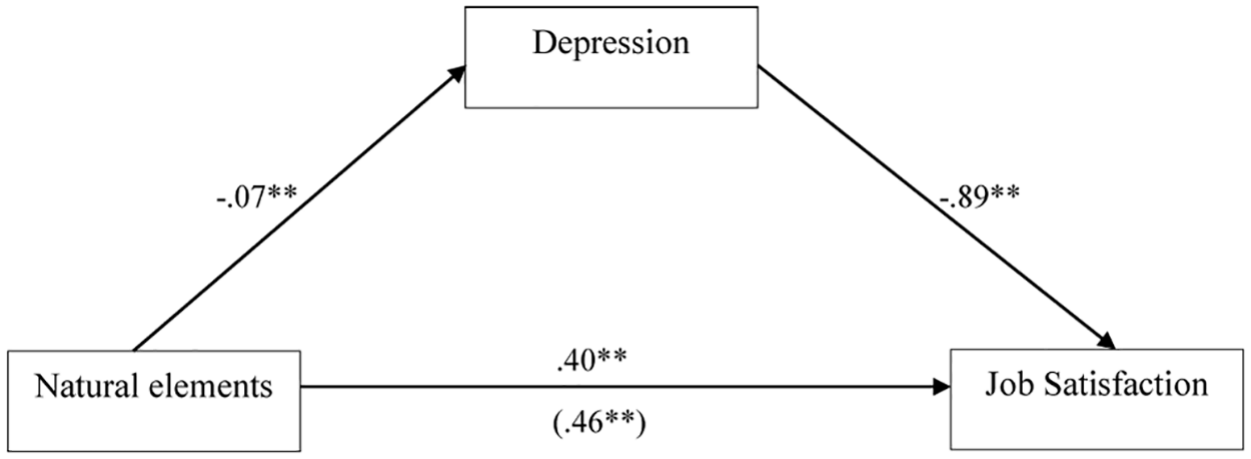
Mediating effects of depression between natural elements and job satisfaction. Values indicate the standardized regression coefficients taking into account the effects of control variables. A value in parenthesis indicate the bivariate coefficient between natural elements and job satisfaction.

We used Hayes’ PROCESS analysis [68] to test for the mediating effect of depression between natural elements and outcomes (Hypothesis 2). If the 95% confidence interval (CI) does not include zero, it indicates sufficient support of a mediating effect. The results are presented in Table 3 and Fig 1. The mediating effect of depressed mood on the relationship between natural elements and job satisfaction has a point estimate of .06 and a 95% CI of .02—.11; therefore, Hypothesis 2 was supported.

**Table 3:** The mediating effects of depression on the relationship between natural elements and job satisfaction.

Variables	*Product of coefficient*		*Bootstrapping*	
	*B*	*SE*	95% CI lower	95% CI upper
**Total effect**	.46	.06	.34	.57
**Direct effect**	.40	.06	.29	.50
**Indirect effect**	.06	.02	.02	.11

Note. N = 401

**Fig 2:**
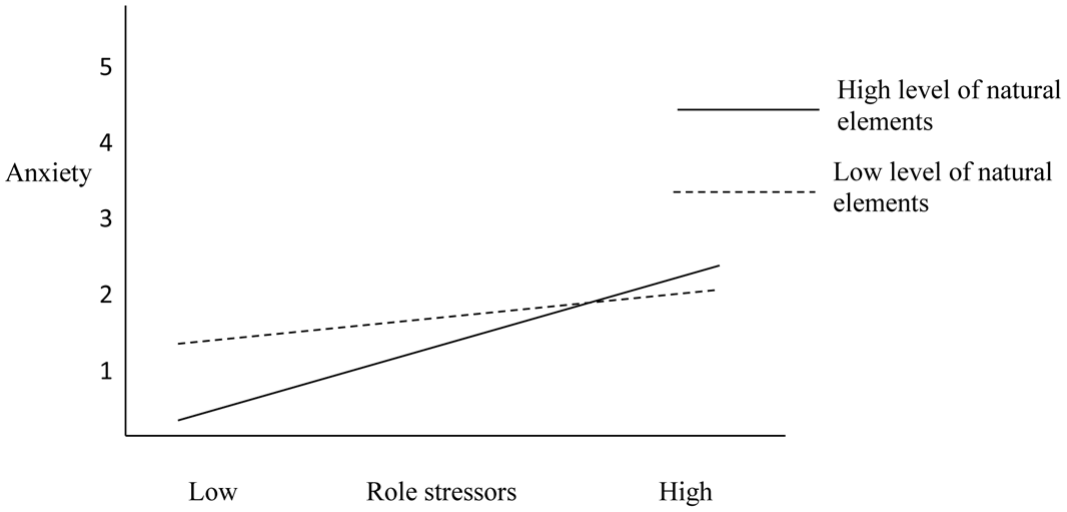
Natural elements exposure as a moderator of the relationship between role stressors and anxiety.

**Fig 3:**
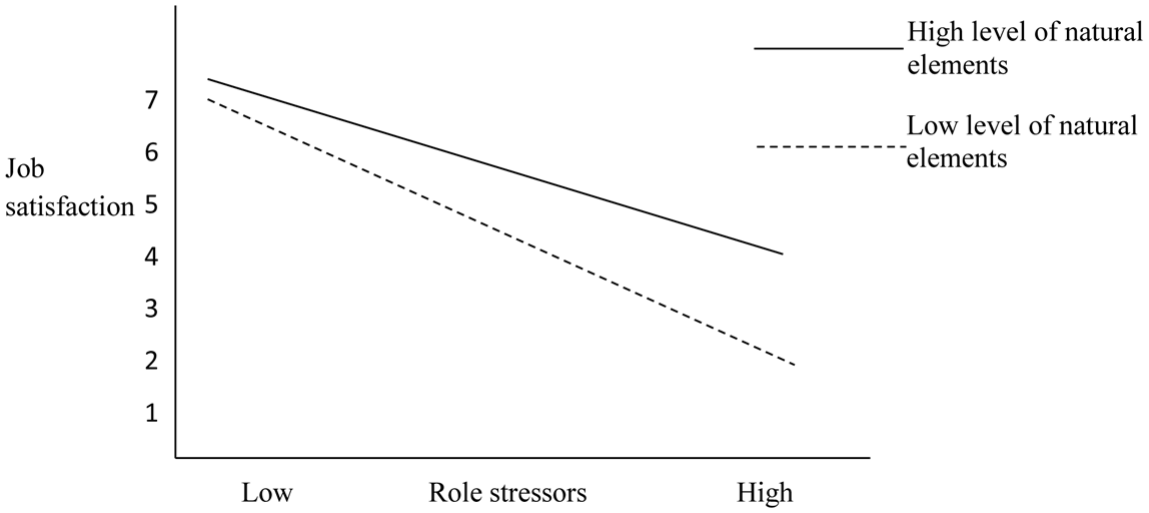
Follow-up for natural elements as a moderator of the relationship between role stressors and job satisfaction.

We used hierarchical regression analysis to test Hypotheses 3, stating natural elements would moderate the relationship between role stressors and employee mental health (a) and work attitudes (b). The results are displayed in Table 4. Natural elements exposure moderated the relationship between role stressors and anxiety (*β* = .17, *p* < .01), but not depressed mood, thereby partially supporting Hypothesis 3a. The moderating effects of natural elements were plotted using mean splits (Fig 2). These results indicated that the relationship between role stressors and anxiety was weaker for individuals who were exposed to more natural elements than their counterparts. Natural elements exposure also moderated the relationship between role stressors and job satisfaction (*β* = .13, *p* < .01), but not organizational commitment, again partially supporting Hypothesis 3b. The moderating effects of natural elements were plotted using mean splits (Fig 3), with results suggesting that the relationship between role stressors and job satisfaction varied based on the level of natural elements exposure. In other words, for individuals with greater exposure to natural elements, the relationship between role stressors and job satisfaction was weaker than those with less exposure to natural elements.

**Table 4:** The moderating effects of natural elements on outcome variables.

Criteria	Variables	Step 1 *β*	Step 2 *β*	Step 3 *β*	Δ *R*^*2*^	Total *R*^*2*^
**Depression**	Sex ^a^	-.05	-.04	.03	.02**	.25**
	Age	-.15**	-.12*	-.12*		
	Natural elements (N)		.44**	.46**	.22**	
	Role stressors (R)		-.14**	-.14**		
	N * R			.07	.00	
**Anxiety**	Sex	-.17**	-.15**	-.13*	.04**	.20**
	Age	-.13**	-.09	-.08		
	Natural elements (N)		-.01	-.00	.13**	
	Role stressors (R)		.37**	.41**		
	N * R			.17**	.03**	
**Job satisfaction**	Sex	-.08	-.07	-.06	.01	.30**
	Age	.03	.01	.01		
	Natural elements (N)		.34**	.34**	.15**	
	Role stressors (R)		-.40**	-.36**		
	N * R			.13**	.02**	
**Organizational commitment**	Sex	-.02	.03	-.04	.01	.31**
	Age	.12*	.08	.08		
	Natural elements (N)		-.46**	-.48**	.30**	
	Role stressors (R)		.27**	.27**		
	N * R			-.06	.00	

Note. N = 383–403.

^a^ coded 1 = male, 2 = female.

*p < .05,

**p < .01

Hypothesis 4 stated that sunlight exposure (i.e., direct sunlight and indirect sunlight) would be negatively related to depressed mood and anxiety and positively related to work attitudes. It was examined with hierarchical regression, and the results are presented in Tables 5 and 6. Direct sunlight was positively related to anxiety (*β* = .21, *p* < .01), job satisfaction (*β* = .20, *p* < .01), and organizational commitment (*β* = .18, *p* < .01). Indirect sunlight was negatively related with depressed mood (*β* = -.20, *p* < .01) and positively related with organizational commitment (*β* = .36, *p* < .01). Hypothesis 4 was partially supported.

**Table 5:** Effects of direct sunlight exposure on outcome variables.

Criteria	Variables	Step 1 *β*	Step 2 *β*	Δ *R*^*2*^	Total *R*^*2*^
**Depression**	Sex ^a^	-.05	-.04	.03**	.03**
	Age	-.16**	-.18**		
	Direct Sunlight		.06	.00	
**Anxiety**	Sex	-.18**	-.14**	.05**	.10**
	Age	-.16**	-.20**		
	Direct Sunlight		.21**	.04**	
**Job satisfaction**	Sex	-.06	-.03	.00	.04**
	Age	.04	.00		
	Direct Sunlight		.20**	.04**	
**Organizational commitment**	Sex	-.02	.02	.02*	.05**
	Age	.13**	.10*		
	Direct Sunlight		.18**	.03**	

Note. N = 397–421.

^a^ coded 1 = male, 2 = female.

*p < .05.

**p < .01

**Table 6:** Effects of indirect sunlight exposure on outcome variables.

Criteria	Variables	Step 1 *β*	Step 2 *β*	Δ *R*^*2*^	Total *R*^*2*^
**Depression**	Sex ^a^	-.03	.05	.02**	.06**
	Age	-.15**	-.15**		
	Indirect Sunlight		-.20**	.04**	
**Anxiety**	Sex	-.17**	-.17**	.05**	.06**
	Age	-.14**	-.14**		
	Indirect Sunlight		-.07	.00	
**Job satisfaction**	Sex	-.07	-.04	.00	.17**
	Age	.02	.03		
	Indirect Sunlight		.40**	.16**	
**Organizational commitment**	Sex	-.03	.05	.01	.14**
	Age	.11*	.11*		
	Indirect Sunlight		.36**	.13	

Note. N = 397–421.

^a^ coded 1 = male, 2 = female.

*p < .05,

**p < .01

We used dominance analysis to disentangle the effects of natural elements from sunlight (that is, to examine the relative amounts of variance accounted for by each). Dominance analysis, also called relative weights analysis, compares all possible *R*^*2*^ values accounted for by every possible combination of the predictors and describes the relative impact that each variable has on the overall *R*^2^ [69]. The results of the dominance analyses are presented in Tables 7 and 8. The average *R*^*2*^ values are shown in Table 7, and the relative contributions of natural elements, direct sunlight, and indirect sunlight on the outcome variables are displayed in Table 8.

**Table 7:** Dominance analysis: average *R*^*2*^ Values.

DV	K	Natural elements	Direct Sunlight	Indirect Sunlight
**Depression**	0	.03	.00	.04
	1	.02	.01	.03
	2	.00	.01	.03
**Anxiety**	0	.00	.04	.01
	1	.03	.05	.01
	2	.00	.06	.02
**Job satisfaction**	0	.13	.04	.16
	1	.08	.01	.09
	2	.04	.00	.04
**Organizational commitment**	0	.09	.03	.13
	1	.05	.00	.07
	2	.02	.00	.07

Note. N = 383–403. K refers to the number of predictors, excluding a variable in column. For example, K = 0 indicates no other variable was entered into the equation (see [69]).

**Table 8:** Dominance analysis: relative amounts of variance and total *R*^*2*^ values.

DV	Natural elements	Direct Sunlight	Indirect Sunlight	Total *R*^*2*^
**Depression**	29.82%	14.91%	55.26%	.06
**Anxiety**	3.19%	78.19%	18.63%	.07
**Job satisfaction**	42.77%	6.80%	50.43%	.20
**Organizational Commitment**	37.35%	6.33%	56.33%	.14

Note. N = 383–403.

The overall *R*^*2*^s accounted for by the three predictors were .06 for depression, .07 for anxiety, .20 for job satisfaction, and .14 for organizational commitment. Indirect sunlight was the dominant predictor of all outcomes except anxiety, which was dominantly predicted by direct sunlight. Direct sunlight explained 78.19% of the overall *R*^*2*^ in anxiety, whereas indirect sunlight explained 55.26% of the overall *R*^*2*^ in depression, 50.43% of the overall *R*^*2*^ in job satisfaction, and 56.33% of the overall *R*^*2*^ in organizational commitment.

## Discussion

This study examined how natural elements and sunlight exposure in workspaces influence employee mental health and job attitudes. Both natural elements and sunlight exposure influenced employee mental health and job attitudes. Natural elements and sunlight exposure simultaneously explained more variance in job attitudes than mental health outcomes (*R*^2^s of .20 and .14 versus .06 and .07).

Sunlight had a more powerful effect than natural elements. It had considerably stronger effects on mental health outcomes, with indirect sunlight associated with about twice as much of the explained variance in depression as natural elements exposure (55.26% versus 29.82%). The disparity was even larger with anxiety. Indirect sunlight was associated with more variance in job attitudes, although disparity was not as large as it was with mental health outcomes.

We found that greater levels of natural elements exposure were associated with lower depressed mood and higher job satisfaction and organizational commitment (Hypothesis 1). Contrary to our expectations, natural elements exposure did not have a direct effect on anxiety, though it did seem to have a buffering effect (Hypothesis 3)—mitigating some effects of role stressors on anxiety. Although natural elements exposure did not buffer the relationship between role stressors and depressed mood, it moderated the relationship between role stressors and job attitudes. The relationships between role stressors and job satisfaction or organizational commitment were weaker for individuals with greater exposure to natural elements (Hypothesis 3). The ameliorating effects of natural elements on this relationship may be explained in several ways. First, natural elements exposure may buffer the relationship by counteracting the effects of stressors on mood, which would influence job attitudes. Alternatively, it might be a *byproduct* of factors associated with exposure to natural elements. For example, greater exposure to natural elements at work may also be associated with greater autonomy and status, often associated with satisfaction and commitment. Another possibility is that people who work outdoors (e.g., landscapers, construction workers) not only have more exposure to natural elements but also engage in more physical activity, which can improve attitudes and mental health.

We also found that the relationship between natural elements exposure and job satisfaction is mediated by depressed mood (Hypothesis 2). This is a unique and potentially important finding. First, this finding suggests that natural elements exposure influences mood and that mood in turn influences job satisfaction. Few studies in organizational psychology have explicitly examined the relationship between depressed mood and job satisfaction, although many typically include both constructs as dependent variables. A meta-analytic study by Faragher et al. found a fairly robust relationship between job satisfaction and depression (corrected correlation *ρ* = .42, *k* = 46), and their interpretation was that job dissatisfaction leads to depression [70]. This seems reasonable enough—when people are dissatisfied with their jobs, dissatisfaction spills over to mood. Our results, however, indicate that the reverse could also be true. A depressed mood might spill over onto how one experiences a job—with a low mood leading to job dissatisfaction.

Direct and indirect sunlight exposure had different effects on mental health outcomes. Direct sunlight had no effect on depressed mood but was positively related to anxiety. Indirect sunlight was negatively associated with depressed mood, but it had no effect on anxiety. The effect of direct sunlight on anxiety was unexpected. It may be due to sunlight’s stimulating effects, influencing alertness and vitality [48, 50]. Alternatively, the relationship might have occurred because people who are experiencing anxiety in their workspaces may be more likely to go outdoors to calm themselves or find respite from a situation producing anxiety. Indeed, people with higher levels of arousal are more likely to immerse themselves to nature [71]. The opportunities that anxious employees have to go outdoors into nature or sunlight—and the effects of those opportunities—are intriguing areas for future research.

As was the case with natural elements exposure, sunlight exposure was positively related to job satisfaction and organizational commitment. Perhaps the most promising theoretical explanations are conservation of resources theory [37] and social exchange theory [38]. Conservation of resources theory argues that people who obtain resources they value are likely to experience less stress and more satisfaction. This is not dissimilar to Locke’s theory of job satisfaction [72], which argues that job satisfaction results from a job meeting expectations for what a person values. To the extent that natural elements and sunlight exposure are valued resources, it stands to reason that they will be associated with greater job satisfaction and possibly organizational commitment. Social exchange theory [38] might be a better explanation for the association with organizational commitment. People who perceive that their organizations provide valued resources or take care of them are more likely to feel an obligation to pay the organization back [73]. One way of doing this is by evidencing greater organizational commitment [74].

### Limitations

We used a cross-sectional design, which precludes the ability to draw causal inferences. We also used self-reported, single source data. Although some suggest that this can lead to common method variance (CMV) [75], others argue that concerns over CMV may be overemphasized [75–78] and that in-depth analyses can help overcome the potential limitations associated with CMV [75, 79]. In this vein, we found that our results were generalizable across a bi-national sample and that our scales displayed similar internal properties within each sample. Of course future research should seek to replicate these findings using additional measures and on other samples.

### Policy Implications

Although some organizations are creating more naturalistic environments for their employees, this area remains under examined [80, 81]. This present study adds to the literature suggesting that natural elements and sunlight exposure have positive effects on employee mental health and job attitudes, thereby supporting policies that encourage the design of workspaces with natural elements and sunlight exposure.

Remodeling workspaces can be expensive. However, there are less costly yet effective design and policy approaches for enhancing exposure to natural elements and sunlight in the workplace [82]. For example, organizations could allow employees to keep plants in their offices or hang photos of nature on office walls, and allow employees time for walks outside of the office. These small and inexpensive changes could result in noticeably better mental health and work attitudes. These results suggest that organizations and policy makers should pay more attention to the physical design of workspaces.

## Supporting Information

S1 Scale Exposure to natural elements. (PDF)

S2 Scale Exposure to sunlight. (PDF)
