# Supplementary material for: Why We Need More Nature at Work: Effects of Natural Elements and Sunlight on Employee Mental Health and Work Attitudes
Source: PLoS One. 2016 May 23;11(5):e0155614. doi: 10.1371/journal.pone.0155614 (PMC4877070; doi:10.1371/journal.pone.0155614)
Supplement: S1 Scale — (PDF) [file pone.0155614.s001.pdf]

Scale 1. Exposure to Natural elements.

Instruction: Please respond to the following statements about your workplace:

Natural elements exposure	Strongly disagree	Disagree	Neither agree nor disagree	Agree	Strongly agree
1. There are potted plants in my workspace.	☐	☐	☐	☐	☐
2. I am exposed to depictions of nature (paintings, photographs) at my workspace	☐	☐	☐	☐	☐
3. I view pictures of greenery on my computer (screensaver, saved pictures).	☐	☐	☐	☐	☐
4. There are images of greenery (realistic photographs, paintings) in my workspace.	☐	☐	☐	☐	☐
5. I look at realistic images of greenery at work (realistic photographs, paintings).	☐	☐	☐	☐	☐
6. There are enough realistic depictions of greenery (photographs or realistic paintings) in my workspace.	☐	☐	☐	☐	☐
7. Overall, I am satisfied with the amount of greenery I am exposed to in my workspace.	☐	☐	☐	☐	☐
8. There are windows at my workspace with views of greenery (trees, plants, flowers).	☐	☐	☐	☐	☐
9. In general, my workspace features enough greenery.	☐	☐	☐	☐	☐
