# Supplementary material for: Why We Need More Nature at Work: Effects of Natural Elements and Sunlight on Employee Mental Health and Work Attitudes
Source: PLoS One. 2016 May 23;11(5):e0155614. doi: 10.1371/journal.pone.0155614 (PMC4877070; doi:10.1371/journal.pone.0155614)
Supplement: S2 Scale — (PDF) [file pone.0155614.s002.pdf]

Scale. Exposure to Sunlight.

Instruction: Please respond to the following statements about your workplace:

Sunlight exposure	Strongly disagree	Disagree	Neither agree nor disagree	Agree	Strongly agree
1. I am exposed to sunlight through windows in my workspace.	☐	☐	☐	☐	☐
2. Overall, I am satisfied with the amount of sunlight exposure I get at work.	☐	☐	☐	☐	☐
3. I have control over how much sunlight comes into my workspace (control over windows, blinds, skylights).	☐	☐	☐	☐	☐
4. There are windows that allow for natural sunlight to come into my workspace.	☐	☐	☐	☐	☐
5. I get enough sun exposure while I am at work.	☐	☐	☐	☐	☐
6. I am exposed to direct sunlight (from being outside) while at work.	☐	☐	☐	☐	☐
7. I have control over how often I can go outside and get direct sunlight exposure while I am at work.	☐	☐	☐	☐	☐
8. I make time to go outdoors and be in the sun while I am at work.	☐	☐	☐	☐	☐
